# Umbelliprenin Increases the M1/M2 Ratio of Macrophage Polarization and Improves the M1 Macrophage Activity in THP-1 Cells Cocultured with AGS Cells

**DOI:** 10.1155/2021/9927747

**Published:** 2021-07-13

**Authors:** MohammadTaher Bahrami, Mostafa Haji Molla Hoseini, Mitra Rezaei, Seyed Ali Ziai

**Affiliations:** ^1^Department of Pharmacology, School of Medicine, Shahid Beheshti University of Medical Sciences, Tehran, Iran; ^2^Department of Immunology, School of Medicine, Shahid Beheshti University of Medical Sciences, Tehran, Iran; ^3^Medical Nanotechnology and Tissue Engineering Research Center, Shahid Beheshti University of Medical Sciences, Tehran, Iran; ^4^Department of Pathology, School of Medicine, Shahid Beheshti University of Medical Sciences, Tehran, Iran

## Abstract

**Background:**

Gastric adenocarcinoma is the fifth most diagnosed malignancy in the world. The immune system consists of a heterogeneous mixture of macrophages that defense the body through phagocytosis and the production of different cytokines and chemokines. Tumors cause macrophages to polarize differently in the manner of their favorite growth and angiogenesis. Umbelliprenin, a natural sesquiterpene coumarin, has been shown to have anticancer properties against some tumors, including gastric adenocarcinoma. The aim of our study was to investigate the effect of umbelliprenin on the polarization of macrophages in addition to the measurement of some of the soluble factors they produce.

**Method:**

The values of IC_5_ and IC_50_ for umbelliprenin in the AGS and THP-1 cells were estimated using the MTT assay. THP-1 cells were treated with 10 *μ*M umbelliprenin, either alone or cocultured with AGS cells. Flow cytometry analysis of treated THP-1 cells was performed for CD68, CD86, and CD206 markers to evaluate M0, M1, and M2 macrophages polarization, respectively. AGS cells were assessed for apoptosis and necrosis by flow cytometry after labeling with Annexin V-FITC and propidium iodide. Interleukin- (IL-) 10 and IL-12 contents were measured in the supernatant by the ELISA method. Griess Reaction assay technique was used to determine nitric oxide (NO) concentration.

**Results:**

The results of the MTT showed lower toxicity of umbelliprenin in THP-1 (IC_50_ = 75.79) compared to the AGS cell line (IC_50_ = 48.81). Umbelliprenin significantly increased the M1/M2 ratio. IL-10 content decreased significantly in the supernatant of M1 and M2 cells after umbelliprenin treatment, while IL-12 increased in the supernatant of M1 cells and decreased in the supernatant of the M2 cells. Umbelliprenin caused an increase in the NO in the supernatant of the M1 cells.

**Conclusion:**

Umbelliprenin alters the macrophage's secretions and its phenotypes in favor of tumor suppression.

## 1. Introduction

Gastric adenocarcinoma (GA) is the fifth most commonly diagnosed and the most malignancy tumor of the stomach. Although the prevalence of cancer is decreasing in industrialized countries, it is still the third leading cause of cancer-associated mortalities. Unfortunately, up to now, there is not a lot of therapeutic options. The surgical resection is considered a potentially curative option in the early stages. However, the result is significantly influenced by the procedure aggressiveness, recurrence, and distant metastasis [1]. Conventional treatment, also known as neoadjuvant and adjuvant chemotherapy, could improve prognosis and overall survival, but the intrinsic and acquired drug resistance may reduce therapeutic success [2].

The Inflammatory Microenvironment plays an important role in the progression of cancer. Macrophages as inflammatory cells participate in the process of innate immunity, inflammation, and a major part of the leukocyte infiltrate present in solid tumors [3, 4]. Because of the plasticity of macrophages, there are at least two subsets of monocytes in human blood, based on classical or alternative activation processes. Inflammatory stimuli, such as lipopolysaccharides (LPS) and interferon-*γ* (IFN-*γ*), produce classically activated macrophages, also known as M1 macrophages, while anti-inflammatory stimuli, such as IL4 and IL13, polarize macrophages toward its alternatively activated form, also known as M2 macrophages. Each type has specific receptor expression, cytokine, chemokine production, and therefore specific functions. The M1 subset exhibits proinflammatory cytokines such as interleukin- (IL-) 12, which promote the Th1 effector response while the anti-inflammatory M2 macrophages are characterized by higher IL-10 and lower IL-12 production profiles [5–7].

Tumor-associated macrophages (TAMs) represent the largest population of infiltrating inflammatory cells in malignant tumors, altering the tumor microenvironment to contribute to the regulatory process of tumor progression through immunosuppression, stroma formation, invasion, angiogenesis, metastases, and secretion of proangiogenic factors such as vascular endothelial growth factor (VEGF). Functional states of the TAMs constantly shift in response to changes in the tumor microenvironment. Within the tumor cells, M2-polarized TAMs were detected predominantly which affects the efficacy of anticancer drugs [8].

**Figure 1:**
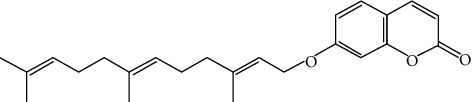
Structure of umbelliprenin.

Umbelliprenin (UMB) is a natural sesquiterpene coumarin compound and has a similar structure to aurapten, an antitumor compound that is synthesized by various *Ferula* species, such as *Citrus limon* (Figure 1) [9]. UMB is the effective component of *Ferula sinkiangensis*, a traditional Chinese medicine (TCM) that has been used in the Xinjiang District for centuries to treat stomach disorders [10]. UMB has been shown to possess distinctive pharmacological and biological characteristics such as anti-inflammatory, immunomodulatory, antitumor, antioxidant, cytotoxic, antibacterial, anti-HIV, antileishmanial, and antiosteoporosis properties [11, 12].

The anticancer properties of UMB are mediated through extrinsic and intrinsic apoptotic pathways [13], which cause inhibition of the G0/*G*1 cell cycle and reduction of tumor migration and invasion. This process can be done through Wnt, Bax, NF-*k*B, cyclone *E*, and some other pathways [14].

This study was aimed to investigate the effect of UMB on the polarization of macrophages and its effect on TAMs using the AGS cell line cocultured with THP-1.

## 2. Materials and Methods

### 2.1. Reagents

Umbelliprenin (UMB) was bought from Golexir pars Co. (Mashhad-Iran). RPMI-1640 medium, 3-(4,5-dimethylthiazol-2-yl)-2, 5-diphenyltetrazolium bromide (MTT) powder, and phorbol-12-myristate-13 acetate (PMA) were purchased from Sigma-Aldrich (St. Louis, MO, USA). Fetal bovine serum (FBS) and penicillin/streptomycin (100 U/mL) were purchased from (Gibco®, Life Technologies, USA), and Trypan blue and DMSO were purchased from Merck (Germany).

### 2.2. UMB Preparation

The stock solution was prepared by dissolving 2 milligrams of UMB in 25 *μ*L DMSO to form a 218.3 mM solution, and a serial dilution was made. We added 0.5 *μ*L of UMB solution to each 200 *μ*L of well, making the final nontoxic concentration of DMSO (less than 0.25% *v*/*v*) in all samples.

### 2.3. Cell Culture and MTT Assay

THP-1 and AGS cell lines have been obtained from the Pasteur Institute (Tehran-Iran). The AGS and THP-1 cell lines were both cultivated in the RPMI-1640 medium complemented by 10% FBS, 100 U/ml penicillin, and 100 *μ*g/ml streptomycin, which were incubated at 37°, 95% humidified, and 5% C0_2_. Two cell lines were passaged at least five times before use.

Exponentially growing THP-1 (10000 cells/well) and AGS (4000 cells/well) isolates were seeded in triplicate in 96-well plates containing 200 *μ*L of RPMI-1640 medium incubated for 24 hours (37°C, 95% humidity, 5% CO_2_). The next day, the medium was replaced and the cells were treated with different concentrations of UMB (2.1, 4.2, 8.5, 17, 34.1, 68.2, 136, 272.8, and 545.7 *µ*M). After 24 hours, 20 *μ*L of MTT (5 mg/ml) solution was added to each well and incubated for an additional 4 hours. The wells containing THP-1 were centrifuged at 1000 rpm and 20°C for 5 minutes in dark conditions before incubation. Then, the medium containing the MTT solution was gently removed, and 100 *μ*L of DMSO was added to each well. After 15 minutes, the absorbance was read at 540 nm and 630 nm using an ELISA reader (BioTek). We used UMB IC_5_ (10 *μ*M) in AGS cells for further investigation.

### 2.4. THP-1 Polarization

To differentiate THP-1 cells from M0 macrophages, PMA (50 nM) was used for 24 hours. Incubation of M0 macrophages with LPS (50 ng/ml) and IFN-*γ* (50 ng/ml) for additional 24 hours led them to become M1-type macrophages. To obtain M2-type macrophages, incubation of M0 macrophages was done through the presence of IL4 (25 ng/ml) and IL13 (25 ng/ml) for 24 hours.

### 2.5. Coculture of AGS with THP-1 Derived Macrophage

For the coculture of AGS with M0 macrophages, 4 × 10^5^ THP-1 cells were seeded in each chamber, which was inserted into a 6-well plate containing RPMI 1640 medium treated with 50 nM PMA. Then, for M1 macrophage polarization, LPS (50 ng/ml) and IFN-*γ* (50 ng/ml) were used, or for M2 macrophage polarization, IL4 (25 ng/ml) and IL13 (25 ng/ml) were added to the medium for 24 hours. Simultaneously, 133 × 10^3^ AGS cells/well were seeded into a 6-well adherent plate containing RPMI 1640 medium. After 24 hours, insert chambers were inserted in the AGS containing wells after changing the medium with a fresh RPMI medium. At 24 hours, the supernatant was used to assess IL-10, IL-12, and nitric oxide. AGS cells were collected to evaluate apoptosis by measuring Annexin V/PI index by flow cytometry. The macrophage subtypes were identified by using CD68 (M0), CD86 (M1), and CD206 (M2) markers via flow cytometry (CyFlow Space).

### 2.6. UMB Treatment Groups

**Figure 2:**
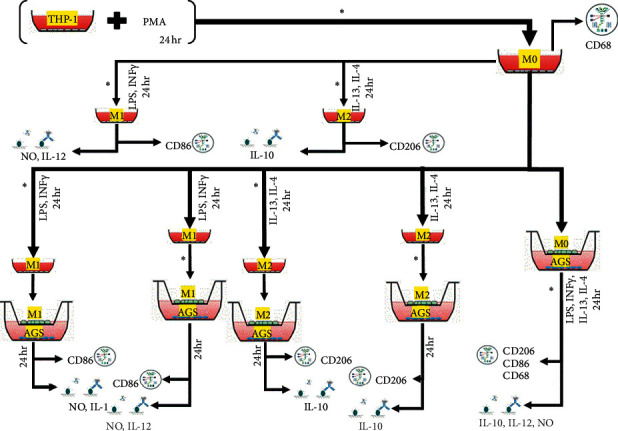
The UMB treatment groups are depicted in detail. UMB IC_5_ (^*∗*^) was introduced in stages. Flow cytometry was used to identify M0, M1, and M2 macrophages using CD68, CD86, and CD206. ELISA was used to assess the levels of IL-10 and IL-12. Griess Reaction assay technique was used to determine nitric oxide (NO) concentration.

In the presence of PMA, THP-1 cells differentiate into M0 macrophages. M0 macrophages could be polarized into M1 or M2 macrophages by adding LPS + IFN- or IL4 + IL13 to the culture media. UMB was added to the culture media at various stages of macrophage proliferation to assess the effects on macrophage polarization as well as IL-10, IL-12, and nitric oxide concentration changes in the culture media as a macrophage functionality indicator (Figure 2).

### 2.7. Nitric Oxide Assay

The evaluation of nitrite as a marker for nitric oxide was carried out through the Griess reagents to measure nitric oxide. To perform the test, 100 *μ*L cell culture supernatants were collected and mixed with 50 *μ*L sulfanilamide 1% and 50 *μ*L N-1-naphthyl ethylene dihydrochloride 0.1%. After 10 minutes of incubation at room temperature, absorbance was measured at 540 nm using the ELISA reader.

### 2.8. Interleukin Assay

All reagents and antibodies for IL-10 and IL-12 cytokines were purchased from BD Biosciences Co. and tested according to the protocol of the kit. The absorbance was measured at 450 nm with the ELISA reader.

### 2.9. Annexin V/PI Assay

In order to address the extent of apoptosis and necrosis of AGS cells, Annex V-FITC/PI dual staining was performed using FITC Annex V Apoptosis Detection Kit I, BD, USA. The trypsin solution that contained EDTA (0.02%) and trypsin (0.05%) in PBS was used for the harvesting of AGS cells. Then, the cells were washed three times with a PBS solution and resuspended in a 1X binding buffer at a concentration of 1 × 10^6^ cells/ml. 100 *μ*L was transferred to the culture tube, and 5 *μ*L FITC Annex V and 5 *μ*L PI were added. The mixture was incubated in the dark for 15 minutes. In the end, 400 *μ*L of 1X binding buffer was added to each tube, and the flow cytometry (Beckman Coulter Epics XL.MCL) analysis was performed.

### 2.10. Data Analysis

Graphing of charts and statistical comparison of group means and measurement of IC_50_ and IC_5_ of the MTT assay were performed using GraphPad Prism software 6.0, and *P* values <0.05 were considered statistically significant.

## 3. Results

### 3.1. UMB Cytotoxic Effect on AGS and THP-1 Cells

**Figure 3:**
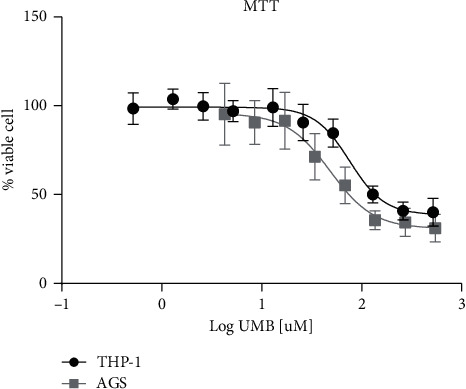
Viability results of THP-1 and AGS cells incubated at different UMB concentrations using the MTT assay. Each point represents the mean ± SD of the triple assay.

The MTT assay was performed to detect UMB cytotoxicity on THP-1 and AGS cell lines over a 24-hour period. The IC_50_ values for THP-1 and AGS cell lines were 75.79 *µ*M (CI 95%; 61.49 to 93.42) and 48.41 *µ*M (CI 95%; 33.02 to 70.97), respectively. The THP-1 cell line value of IC_5_ was 23.26 *µ*M (CI 95%; 15.18 to 35.66), and for AGS, it was 11.12 *µ*M (CI 95%; 3.65 to 33.85). UMB 10 *µ*M as IC_5_ in AGS cells was used in all subsequent experiments to study the effects of UMB on these cells with minimal cytotoxicity (Figure 3).

### 3.2. UMB Effect on Macrophage Polarization

**Figure 4:**
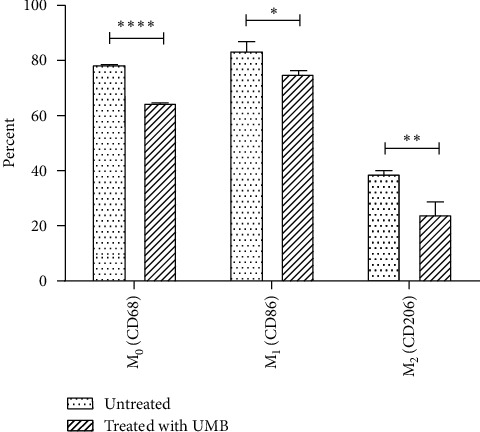
The percentage of M0 (CD68), M1 (CD86), and M2 (CD206) in UMB IC_5_ (10 *μ*M) treated and untreated THP-1 cells was determined by flow cytometry. Each bar represents the mean ± SD of the three separate experiments. ^*∗*^*P* < 0.05; ^*∗∗*^*P* < 0.01; ^*∗∗∗∗*^*P* < 0.0001.

The net effect of UMB IC_5_ on the production of M0, M1, and M2 is a reduction effect that may be related to the cytotoxic effects of UMB (Figure 4).

**Figure 5:**
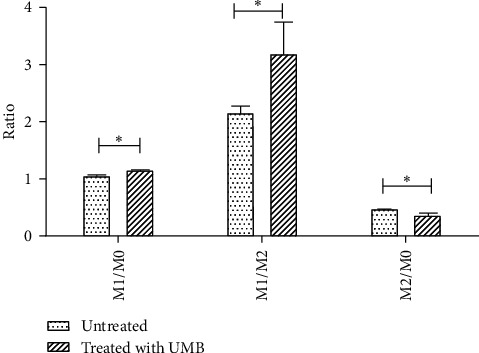
M1/M0, M1/M2, and M2/M0 ratio of UMB-treated macrophages and untreated macrophages cells. Each bar represents the mean ± SD of the three independent experiments. ^*∗*^*P* < 0.05.

The effect of UMB IC_5_ on THP-1 differentiation to M0 macrophage and subsequent polarization to M1 or M2 macrophage subtypes was assessed by comparing the subtype macrophage ratio between UMB-treated and untreated THP-1 cells. The results showed a significant increase in M1/M0 (*P* = 0.0212), a significant decrease in M2/M0 (*P* = 0.0334), and a significant increase in M1/M2 (*P* = 0.0483), indicating that UMB-treated cells were acting in favor of M1 dominance and M2 reduction (Figure 5).

### 3.3. UMB Effect on AGS Cocultured with THP-1 Cells

**Figure 6:**
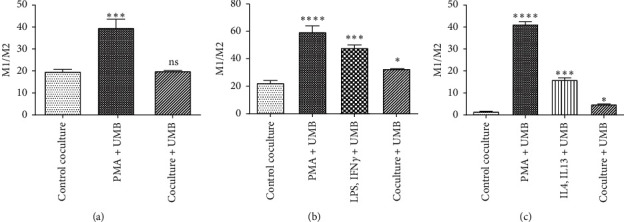
The M1/M2 ratio of cocultured UMB IC5 (10 *μ*M) treated macrophages with AGS cells was determined by flow cytometry in the M0 (a), M1 (b), and M2 (c) polarization processes. Each bar represents the mean ± SD of the three independent experiments. ^*∗*^*P* < 0.05; ^*∗∗∗*^*P* < 0.001; ^*∗∗∗∗*^*P* < 0.0001; ns: not significant.

THP-1 cells were seeded into insert chambers and differentiated into M0, M1, or M2 subtype macrophages by adding differentiating factors. UMB + PMA significantly increased the M1/M2 compared to the control group, but the UMB M1-potentiating effect decreased after M0 differentiation in the coculture + UMB group (Figure 6(a) and Table 1). In the M1 polarization pathway, UMB + LPS and IFN-*γ* increased the M1/M2 ratio (Figure 6(b) and Table 1), and in the M2 polarization, UMB + IL4 and IL13 increased this ratio significantly too (Figure 6(c) and Table 1). UMB also significantly increased the ratio of M1/M2 when added to the polarized M1 or M2 cells (Figures 6(b) and 6(c) and Table 1).

### 3.4. Apoptosis/Necrosis Analysis of AGS Cells

**Figure 7:**
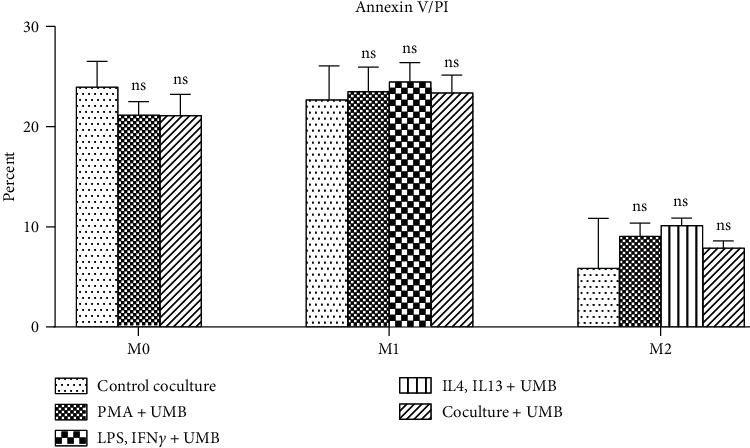
Annexin V/PI assay of AGS cells cocultured with UMB 10 *μ*M treated M0, M1, and M2 cells. Each bar represents the mean ± SD of the three independent experiments. ^*∗*^*P* < 0.05; ^*∗∗*^*P* < 0.01; ^*∗∗∗*^*P* < 0.001; ^*∗∗∗∗*^*P* < 0.0001; ns: not significant.

In order to assess the potential apoptotic and necrosis potentiation of UMB in cocultured AGS cells, cocultured AGS cells were analyzed via Annexin V-FITC/PI double staining flow cytometry after 24 hours of UMB 10 *µ*M exposure. Our results showed no statistically significant change in the fraction of Annexin V/PI in AGS cells cocultured with M0, M1, and M2 cells after the addition of UMB (Figure 7).

### 3.5. UMB Effect on Nitric Oxide Production

**Figure 8:**
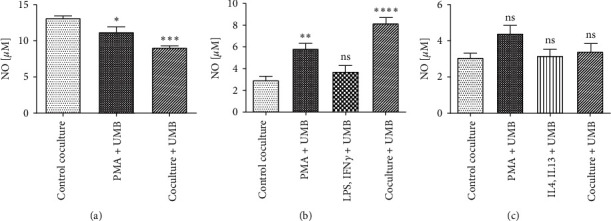
Nitric oxide assay in the supernatant of UMB 10 *μ*M treated macrophages with AGS cells in M0 (a), M1 (b), and M2 (c) coculture. Each bar represents the mean ± SD of the three independent experiments. ^*∗*^*P* < 0.05; ^*∗∗*^*P* < 0.01; ^*∗∗∗*^*P* < 0.001; ^*∗∗∗∗*^*P* < 0.0001; ns: not significant.

In the present study, nitrite was considered as a nitric oxide production index. Cocultured supernatant media were evaluated using a nitric oxide assay kit. The results showed that UMB significantly decreased NO production in the M0 PMA + UMB and coculture + UMB groups (Figure 8(a), and Table 2) but increased NO production in the M1 cocultured macrophages while having no significant effect on the LPS + IFN-*γ* + UMB group (Figure 8(b), and Table 2). M2 cocultured macrophages did not show a significant difference in the production of nitric oxide (Figure 8(c) and Table 2).

### 3.6. UMB Effect on Interleukin-10 and Interleukin-12 Production

**Figure 9:**
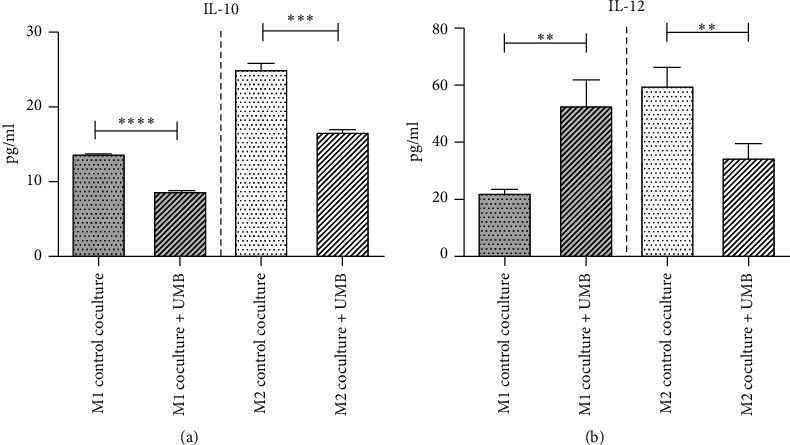
IL-10 and IL-12 concentrations in media of UMB 10 *μ*M treated macrophages cocultured with AGS cells. Each bar represents the mean ± SD of the three independent experiments. ^*∗*^*P* < 0.05; ^*∗∗*^*P* < 0.01; ^*∗∗∗*^*P* < 0.001; ^*∗∗∗∗*^*P* < 0.0001.

The coculture supernatants of M1 and M2 were collected to assay IL-10 and IL-12. The results showed that the contents of IL-10 were significantly decreased in M1 and M2 cocultures. The IL-12 content of the M1 coculture showed a significant increase, although, in the M2 coculture, it decreased significantly (Figure 9 and Table 3).

## 4. Discussion

The antitumor properties of UMB have been studied on various types of cancer cells. In the present experiment, the MTT test showed that The IC_50_ values for THP-1 and AGS cell lines were 75.79 *µ*M (CI 95%; 61.49 to 93.42) and 48.41 *µ*M (CI 95%; 33.02 to 70.97), respectively, which shows lower toxicity of UMB in THP-1 cells.

Since there was not enough information about nontoxic concentrations of UMB, THP-1 cells were treated at AGS IC_5_ concentration of UMB to evaluate its inhibitory effects on cancer cells via modulation of the immune system. The results showed an increase in M1/M2 macrophages ratio during THP-1 differentiation, which is favorable to eradicating cancer cells by the immune system. To ensure if UMB IC_5_ concentration changes an apoptosis or necrosis index in cancer cells, Annexin V/PI was measured, which did not show any significant change in Annexin V/PI index. UMB-induced macrophages functional changes were compared with the control groups by measuring IL-10, IL12, and nitric oxide content of culture media.

Gastric adenocarcinoma is considered a highly aggressive cancer. Less than 30% of patients are expected to survive for 5 years [15]. The cancer prognosis is poor [16], and unfortunately, most cases are diagnosed in advanced stages [1]. Low survival is attributed to the high rates of tumor invasion and metastasis [17]. Various studies have shown that UMB, a compound with a coumarin structure, has anticancerous properties against GL26 (neuroblastoma), A172 (glioblastoma), MCF-7 and 4T1 (breast ductal carcinoma), CT26 and HT29 (colorectal adenocarcinoma) [12], A549 (lung adenocarcinoma), SK-MEL-28 (malignant melanoma), CH1 (lymphoma), M4beu (metastatic pigmented malignant melanoma) [18], HELA (cervix adenocarcinoma), K562 (chronic myelogenous leukemia), and AGS (gastric adenocarcinoma) cells [19]. Previous studies have shown that UMB inhibits gastric tumor growth and migration through inhibition of MMP2 and MMP9. It also reduces Wnt signaling pathway proteins including, Wnt-2, Survivin, *β*-catenin, GSK-3*β*, p-GSK-3*β*, and c-myc [10].

This study was designed considering the differences between the M1 and M2 macrophages in the antitumor immune response [20]. Treatment with different concentrations of UMB (0.5–518 *µ*M) at 24 hours intervals showed lower toxicity on THP-1 cells (*IC*_50_ = 75.79, *IC*_5_ = 23.26) in comparison to AGS cells (*IC*_50_ = 48.41, *IC*_5_ = 11.2). Thus AGS IC_5_ (∼10 *μ*M) was chosen to study the effect of UMB on the proliferation of THP-1 cells and its consequences.

In order to ensure that UMB does not have any significant toxic effect at the IC_5_ concentration, Annexin V/PI staining was perfumed. A statistically significant change was not observed in the fraction of Annexin V/PI in AGS cells cocultured with M0, M1, and M2 cells in any stage of proliferation after the UMB treatment.

M1-like macrophages are characterized by high antigen presentation capacity, proinflammatory, microbicidal, and tumoricidal properties. Some of their functions are due to the secretion of reactive oxygen species (ROS), IL-6, IL12, IL-23, and TNF-*α* [21]. M2-like macrophages function in anti-inflammatory, tissue repair and remodeling, parasite clearance, tumor-promoting, and immunoregulatory processes. They support the cancer cells by proceeding angiogenesis via adrenomedullin and secretion of vascular epithelial growth factors (VEGFs) while its immunosuppression function is mediated by expression of IL-10, PD-L1 (programmed death-ligand 1), and TGF*β* [22].

Tumor microenvironment (TME) alters macrophage polarization predominantly to the M2-like type by producing a variety of cytokines, growth factors, and other molecules [7], which results in drug resistance and radioprotective effect and subsequent therapeutic failure [23]. It also amplifies tumor aggressiveness through invasion, progression, and metastases [24]. Higher densities of TAMs have been observed in more advanced gastric cancer tumor stages [25].

Previous studies have mentioned the biological relevance of the M1/M2 ratio in cancer prognosis rather than total TAMs macrophages (M1 + M2). The cancer prognosis in the higher ratio M1/M2 tumors is associated with favorable survival outcomes and vice versa [26–28]. Our results showed a slight reduction in macrophages caused by UMB. However, analyzing the UMB effect on THP-1 proliferation showed that the M1/M2 ratio was more affected than the M1/M0 and M2/M0 ratios, which suggests a better cancer prognosis.

To assess the effect of cancer cells on the proliferation of macrophages, AGS cells were cocultured with THP-1 cells. UMB was added simultaneously in one of the THP-1 proliferation phases, including PMA, LPS + IFN-*γ*, IL4 + IL13, or the coculture phase. UMB was added during the PMA-induced M0 differentiation phase, the polarization phase (LPS + IFN-*γ* or IL4 + IL13), and the polarized M1 or M2 cocultured with AGS cells.  Figure 6 shows that UMB increases the M1/M2 ratio with the maximum ratio at the first phase, PMA, and with the minimum ratio at the last phase, cocultured.

Nitric oxide plays a critical role in the development and suppression of tumorigenesis. Performance and outcome of NO are relevant to the concentration and source of NO. While low to moderate NO levels may activate angiogenesis and therefore aggressive phenotypes, higher levels, which could be derived from M1 macrophages, may have an antitumor effect [29].

The macrophage metabolism pathway of arginine is a key point in the promotion or inhibition of cancer cells. The metabolism of arginine to ornithine and urea is activated by M2 macrophages and the produced ornithine could be used in cell repair processes. On the other hand, NO production is activated in M1 macrophages by inducible NO synthase induction (iNOS). This cytotoxic metabolite contributes to the killing power of the M1 macrophages [30]. Previous studies on spleen-separated mononuclear cells that were stimulated with LPS indicated a decrease in iNOS and nitric oxide production while they were treated with more than 10 *µ*M UMB [31]. However, no significant effect on NO production was seen at this concentration in RAW264.7 cells in another study [32]. In the present research, treatment of M0 macrophages cocultured with AGS cells at 10 *μ*M UMB showed a decrease in NO production without any significant difference in M2. However, the M1 cocultured macrophages showed an increase in NO production, which is likely due to an increase in the emergence of M1.

Cytokines are involved in the regulation of most immune responses. IL-12 is known as a proinflammatory cytokine, which acts as a link between the innate and adaptive immune system by inducing the production of IFN-*γ* and polarizing naive CD4 T cells to Th1 [33]. On the contrary, IL-10 is considered to be an anti-inflammatory cytokine that limits excessive inflammation and plays an important role in CD8 T cell maturation. In fact, IL-10 and IL-12 balance immune system response against tumor and autoimmune conditions by antagonizing each other [34].

Treatment of M1 and M2 cocultured macrophages with 10 *µ*M UMB showed that while a significant increase of IL-12 was seen in M1 cocultured medium, it was decreased in M2 cocultured medium; however, IL-10 content was significantly decreased in both M1 and M2 cocultured media. Thus, UMB induced the shifting of macrophage polarization to the M1 subtype and an increase in antitumor function resulted from our findings in this study.

Despite several studies, the effect of umbelliprenin on cancer and the immune system remains unknown, and further research is needed. The authors recommend looking into the impact of umbelliprenin on macrophage-cancer cell direct coculture to see if there is a difference in treated macrophage functionality during direct contact with cancer cells.

## 5. Conclusion

In conclusion, our data showed that umbelliprenin, which has less cytotoxicity on monocytes than the AGS cells, promotes polarization of macrophages to the M1 killer type instead of the M2 supportive type, increases the M1/M2 ratio, and therefore enhances immune system function in countering gastric adenocarcinoma. In addition, UMB affects nitric oxide and IL-12 secretion by M1 macrophages. As a result, it seems UMB supports M1 macrophages and attenuates M2 macrophage functionality; thus, it may be a good candidate as a neoadjuvant therapeutic agent. However, further investigations are required.

## Figures and Tables

**Table 1:** UMB effect on the ratio of M1/M2 in cocultured AGS and THP-1 cells.

	PMA + UMB	LPS + IFN-*γ* + UMB	IL4 + IL13 + UMB	Coculture + UMB	Control
M0-AGS cCoculture	39.88 ± 6.81 (*P* = 0.0009)			20.00 ± 0.53 (ns)	19.93 ± 1.59
M1-AGS coculture	59.97 ± 7.70 (*P* < 0.0001)	48.34 ± 3.74 (*P* = 0.0001)		32.82 ± 0.18 (*P* = 0.0248)	22.57 ± 3.12
M2-AGS coculture	41.47 ± 2.01 (*P* < 0.0001)		16.09 ± 1.57 (*P* < 0.0001)	4.883 ± 0.402 (*P* = 0.0154)	1.65 ± 0.085

All values are the mean ± SD of at least three independent experiments that have been tested in a triplicate. The data analysis was carried out using the ANOVA method.

**Table 2:** Effect of UMB on nitric oxide concentration (*μ*M) in cocultured media.

	PMA + UMB	LPS + IFN-*γ* + UMB	IL4 + IL13 + UMB	Coculture + UMB	Control
M0-AGS coculture	11.30 ± 1.16 (*P* = 0.0229)			9.08 ± 0.39 (*P* = 0.0006)	13.19 ± 0.51
M1-AGS coculture	5.86 ± 0.84 (*P* = 0.0035)	3.74 ± 0.96 (ns)		8.19 ± 1.02 (*P* < 0.0001)	2.97 ± 0.58
M2-AGS coculture	4.41 ± 0.84 (ns)		3.19 ± 0.69 (ns)	3.41 ± 0.84 (ns)	3.08 ± 0.51

All values are the mean ± SD of at least three independent experiments that have been tested in a triplicate. The data analysis was carried out using the ANOVA method.

**Table 3:** UMB effect on interleukin-10 and interleukin-12 concentration (pg/ml) in cocultured media.

	M1-AGS coculture		M2-AGS coculture	
	Coculture + UMB	Control	Coculture + UMB	Control
IL-10	8.77 ± 0.25	13.73 ± 0.12 (*P* < 0.0001)	16.67 ± 0.29	25.07 ± 0.97 (*P* = 0.0001)
IL-12	52.99 ± 9.46 (*P* = 0.0052)	22.29 ± 1.81	34.70 ± 4.90 (*P* = 0.0067)	59.88 ± 6.88

All values are the mean ± SD of at least three independent experiments that have been tested in a triplicate. An unpaired *t*-test was used for data analysis.

## Data Availability

The experimental data used to support the findings of this study are available from the corresponding author upon request.
